# Genetic variation in seed shattering and awn length in a core collection of Japanese local upland rice, *Oryza sativa* L.

**DOI:** 10.1007/s10709-026-00300-8

**Published:** 2026-11-06

**Authors:** Kano Mikumo, Kosuke Yabu, Satoshi Kataoka, Koji Numaguchi, Takashige Ishii, Katsunori Okano, Ryo Ishikawa

**Affiliations:** 1 Laboratory of Plant Breeding, Graduate School of Agricultural Science, Kobe University, 1-1 Rokkodai-Cho, Nada-Ku, Kobe, 657-8501 Japan; 2 Plant Biotechnology Institute, Ibaraki Agricultural Center, Kamikunii, Mito, Ibaraki 311-4203 Japan

**Keywords:** Rice (*Oryza sativa*), Seed shattering, Awn, Upland rice, Core collection

## Abstract

Upland rice is cultivated in dry and rain-fed fields and is grown primarily in regions with limited water resources. Due to the development of irrigation systems and the comparatively lower yield and eating quality of upland rice relative to paddy rice, its cultivated area has decreased in Japan over the years. However, upland rice is known to carry genes controlling important agricultural traits such as drought tolerance and disease resistance. Therefore, they have a large potential serving as genetic resource to improve modern paddy cultivars. Although upland rice is cross-compatible with most paddy rice and shares a close genetic background, its evolutionary history and introduction pathways into Japan remain unclear. In this study, we employed a core collection of Japanese local upland rice to evaluate two domestication-related traits: seed shattering and awn length. Large variations were observed in the degree of seed shattering and awn length. Genetic analyses at known loci controlling these traits indicated that several upland cultivars may carry unidentified loci explaining their seed shattering and awn length. The Japanese local upland rice core collection represents a valuable genetic resource for elucidating genetic alterations in domestication-related traits and clarifying their introduction into Japan.

**Supplementary Information:**

The online version contains supplementary material available at https://doi.org/10.1007/s10709-026-00300-8.

## Introduction

Rice (*Oryza sativa*) is one of the most important food crops, supporting nearly half of the world’s population. There are two rice subspecies, *japonica* and *indica*, that are widely cultivated globally (Oka 1988; Khush 1997; Londo et al. 2006). In addition, *japonica* is further subdivided into tropical and temperate types, and other genetically distinct groups, such as *circum*-aus and *circum*-basmati are also known (Wang et al. 2018; Civáň et al. 2019; Gutaker et al. 2020). Based on their adaptation to water conditions, rice can be classified into upland and lowland ecotypes. Upland rice is well-adapted to rain-fed conditions, whereas lowland rice thrives in well-watered or irrigated conditions (Lyu et al. 2020). Compared with lowland rice, upland rice exhibits lower yield and poorer eating quality. However, through adaptation to its growing environment, upland rice possesses several valuable agricultural traits, such as drought tolerance, that can be used to improve lowland cultivars (Gong et al. 2025).

A major quantitative trait locus (QTL) controlling root growth angle, *DEEPER ROOTING 1* (*Dro1*), has been reported from a cross between the shallow-rooting lowland cultivar IR64 and the deep-rooting upland cultivar Kinandang Patong (Uga et al. 2011). The gene underlying the *Dro1* locus regulates root growth angle downstream of auxin signalling, and this allele contributes to enhanced yield capacity of upland rice under drought conditions (Uga et al. 2013). Additionally, Sun et al. (2022) identified several QTLs related to drought tolerance in upland rice, including *DROUGHT1* (*DROT1*). Genetic analysis of Japanese upland rice exhibiting field resistance to rice blast was conducted, and the *Pi21* gene conferring broad-spectrum resistance was identified (Fukuoka and Okuno 2001). This is an important trait for improving disease resistance in lowland rice, as Fukuoka et al. (2009) performed an introgression of *Pi21* into lowland rice and observed successful field resistance by overcoming a tightly linked locus associated with eating quality.

Upland rice is cross-compatible with lowland rice; however, its origins and distribution in Japan remain unclear. Rice was domesticated from the wild progenitor *O. rufipogon* (Oka 1988). During domestication, repeated cultivation and selection of favourable traits were conducted. Domestication-related traits, such as seed shattering, hull colour, seed dormancy, panicle shape, awn length, grain size, and plant architecture, have been selected to meet human needs. Interestingly, traits such as easy seed shattering and awn are often observed in upland rice; however, knowledge of the genetic basis of these traits is limited. Seed shattering is an advantageous characteristic for wild rice because of its efficient propagation; however, it substantially reduces yield. Consequently, reduced seed shattering was selected as a desirable trait (Ishikawa et al. 2020). Loci involved in reduced seed-shattering behaviour associated with natural variation have been summarised (Ishikawa 2024). The *sh4* locus, encoding a Myb transcription factor, was detected through QTL analysis of the degree of seed shattering between wild rice *O. rufipogon* and an *indica* rice cultivar (Li et al. 2006). Another major locus, *qSH1*, was identified from a cross between the non-shattering cultivar Nipponbare and the moderately shattering cultivar Kasalath (Konishi et al. 2006), with a causal mutation located in the upstream regulatory region of the BEL1-type homeobox gene. A third locus, *qSH3*, was identified in introgression lines carrying Nipponbare chromosomal segments covering *qSH1* and *sh4* (Htun et al. 2014), and the causal mutation was identified in the YABBY gene (Ishikawa et al. 2022).

Another domestication-related trait often observed in upland rice is awn length, characterised by the presence of a bristle-like organ with a barb at the tip of the lemma. Long awns facilitate seed dispersal by promoting the detachment of seeds from the panicle and moving horizontally across the ground or in water (Amarasinghe et al. 2021). However, once non-seed shattering plants are produced through the accumulation of loss-of-function mutations at seed-shattering loci, awns may become unnecessary for harvesting and processing (Ishii and Ishikawa 2018). Several QTLs related to seed awning have been reported in rice. The three major loci are *Awn-1* (*An-1*) (Luo et al. 2013), *LONG AND BARBED AWN1* (*LABA1*)/*Awn-2* (*An-2*) (Hua et al. 2015; Gu et al. 2015), and *REGULATOR OF AWNELONGATION2* (*RAE2*)/*GRAINNUMBER, GRAIN LENGTH AND AWNDEVELOPMENT1* (*GAD1*)/*An-3* (Bessho-Uehara et al. 2016; Jin et al. 2016; Liu et al. 2017). *An-1* encodes a basic helix-loop-helix protein. Two domestication-related non-functional alleles of *An-1* have been identified: a transposon-like InDel in the promoter region (Tn +) and a 1-bp nucleotide G deletion in the second exon (G-), which are commonly observed in temperate/tropical *japonica* and *indica* subgroups, respectively (Luo et al. 2013). *LABA1*/*An-2* encodes the Lonely Guy-like protein 6 (OsLOGL6), a cytokinin-activating enzyme. A 1-bp deletion leading to a frame-shift mutation is frequently observed in tropical *japonica* and *indica* subgroups (Hua et al. 2015; Gu et al. 2015). *RAE2*/*GAD*1/*An-3* encodes EPIDERMAL PATTERNING FACTOR-LIKE 1 (EPFL1), a group of plant-specific secreted peptides. Domesticated alleles of *RAE2* are found in a highly variable GC-rich repeat region of EPFL1, which is characterised by *RAE2*-4C, *RAE2*-5C, and *RAE2*-7C (Bessho-Uehara et al. 2016; Jin et al. 2016; Liu et al. 2017). Although genetic variations at these loci have been reported, their genotypes in upland rice remain unclear.

In Japan, upland rice used to be cultivated in regions with limited water resources (Okano et al. 2005). However, with the improvement of irrigation systems, its cultivation area has declined due to lower productivity. Although the cultivation of upland rice has decreased, it remains a valuable genetic resource for future rice breeding efforts. To effectively utilise and preserve this diverse germplasm, a core collection representing the maximum genetic diversity of Japanese local upland rice has been established using a minimum number of accessions. However, the extent of phenotypic and genetic variation for domestication-related traits within this collection has not yet been comprehensively evaluated. In this study, therefore, we aimed to elucidate the phenotypic variations in seed shattering and awn length using this core collection of Japanese local upland rice, and to characterise the genotypes at loci associated with these traits. This study can potentially provide essential baseline data for understanding the retention of domestication-related traits in Japanese upland rice genetic resources.

## Materials and methods

### Plant materials and growth conditions

The Plant Biotechnology Institute, Ibaraki Agricultural Center, which is responsible for conserving upland rice genetic resources, evaluated 289 upland cultivars for growth and morphology (Okano et al. 2005). Eleven morphological and seven physiological traits were analysed using principal component and cluster analyses, classifying the cultivars into seven groups. Subsequently, a total of 32 upland cultivars that retained 86% of the eleven diversity traits among the original 260 cultivars were selected as the core collection (Okano et al. 2006; Table S1). This will subsequently be referred to as the Japanese local upland rice core collection (URC). Cultivars *O. sativa japonica* cv. Nipponbare, cv. Asahi, *O. sativa indica* cv. IR36, and cv. Kasalath were used as controls in this study. URC and control cultivars were planted in the paddy field at Kobe University during the summer seasons of 2022 and 2023. Specifically, the seed-shattering degree was evaluated in 2022, while the awn length was evaluated in 2023. Seedlings were transplanted to the paddy field in the first week of June with single planting. They were planted in the plot with eight plants per row (20 cm between plants and 25 cm between rows). Among them, three to four well-grown plants of each URC and control cultivar were used to evaluate the degree of seed shattering and awn length. In addition, wild rice *O. rufipogon* acc. W630, which originated in Myanmar, was used for genotyping analyses of seed-shattering and awning loci.

### Measurement of degree of seed shattering

The degree of seed shattering was evaluated by measuring the breaking tensile strength (BTS, presented in gramme-force (gf)), which is the force required to detach a grain from the pedicel, using a digital force gauge (FGP 0.5, Nidec Drive Technology Corporation, Japan). After seed maturation, three panicles were collected from each plant. The BTS values of 25 seeds were measured per panicle, and the average BTS values were calculated. The BTS value for each cultivar was calculated from the average BTS values of three plants.

### Measurement of awn length

In wild rice, awn length varies according to the spikelet position (Ikemoto et al. 2017), and seeds are shed from the panicles soon after grain maturation owing to complete abscission layer formation. Therefore, the first to fifth upper spikelets on the topmost primary branch of the panicle were collected at the heading stage, and their awn lengths were measured based on their positions. To ensure consistent measurements, we specifically selected the largest, fully developed panicles; therefore, the topmost primary branch consistently possessed five or more spikelets across all examined plants, allowing unambiguous identification of the first to fifth spikelets. This focus on the topmost primary branch is well-supported by its strong correlation with other branches in terms of awn patterns (Ikemoto et al. 2017), making it a reliable proxy for the entire panicle. The averages of three panicles were calculated for each plant. Five plants of each cultivar were examined, and the average awn length for each cultivar was calculated.

### Genotyping analysis of known seed-shattering and awning loci

Genotyping analysis was conducted at known loci responsible for reduced degree of seed shattering and awn length. To contextualise the genetic background and human selection history related to regional preferences in Japanese upland rice, the genotype of the *Waxy* locus, which determines the glutinous/non-glutinous starch property, was analysed alongside the loci for seed shattering and awn length. Functional and non-functional alleles were determined using previously developed primers (Table S2, Fig. S1). PCR was conducted using Quick Taq (TOYOBO, Japan) according to the manufacturer’s instructions. Loci containing high GC content such as *sh4*, *An-1* (1-bp deletion), and *RAE2* were amplified using KOD-FX Neo (TOYOBO, Japan), according to the manufacturer’s instructions. In the case of InDel markers of *An-1*, *LABA1*, *RAE2*, and *Waxy*, the PCR products were electrophoresed on a 4% polyacrylamide gel, and banding patterns were visualised using the silver staining method (Panaud et al. 1996). Three loci were analysed using Derived Cleaved Amplified Polymorphic Sequences (dCAPS) markers; the fragments were separated on 2.0% agarose gels after digestion with specific restriction enzymes (Table S2). The gels were stained with Midori Green Direct DNA Stain (NIPPON Genetics, Japan) and the DNA fragments were visualised under blue-green LED light using an FAS-V gel imaging system (NIPPON Genetics, Japan).

## Results

### Evaluation of the degree of seed shattering in URC cultivars

**Fig. 1:**
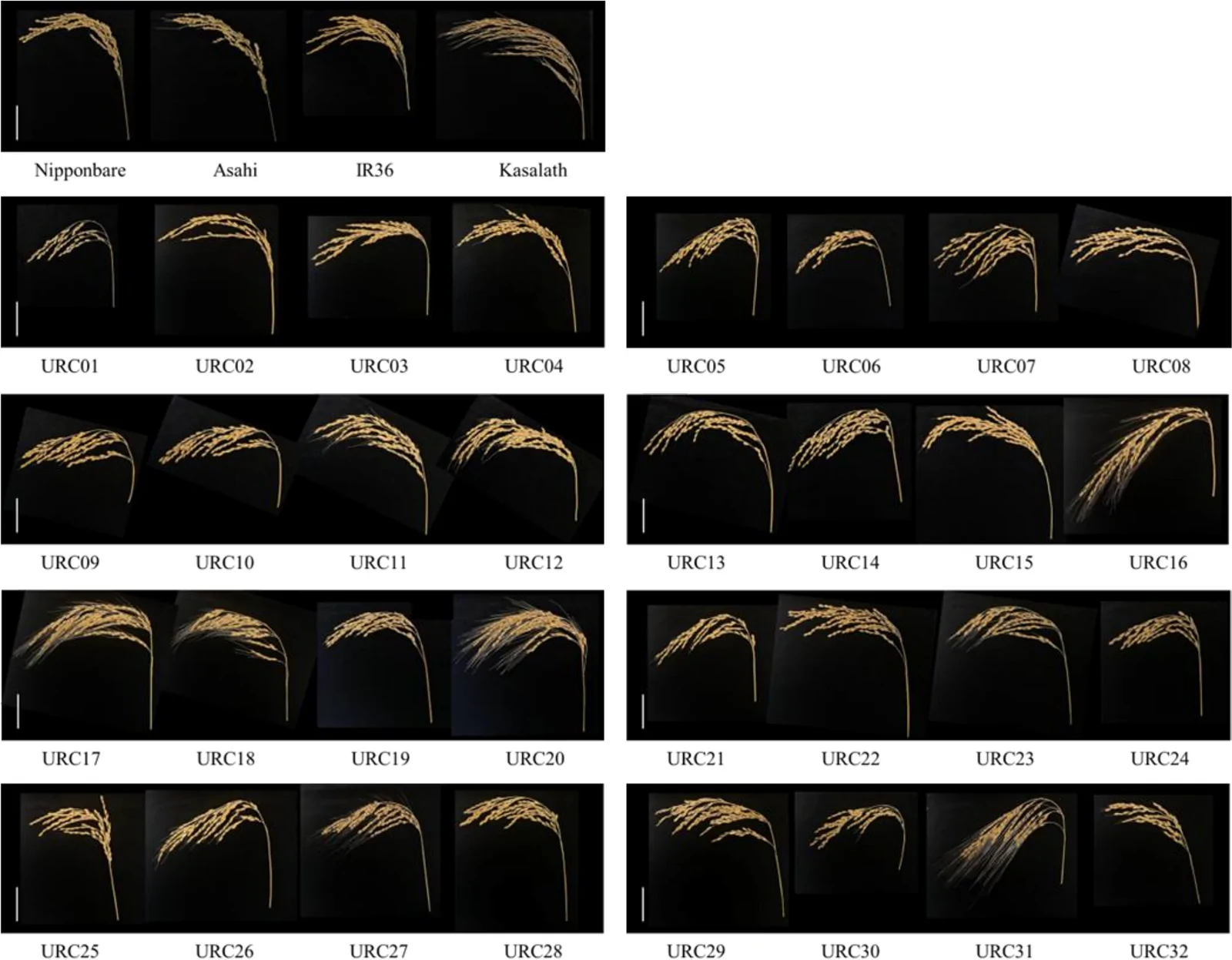
Panicles of the Japanese local upland rice core collection and the control cultivars used in this study. Scale bar = 5 cm

**Fig. 2:**
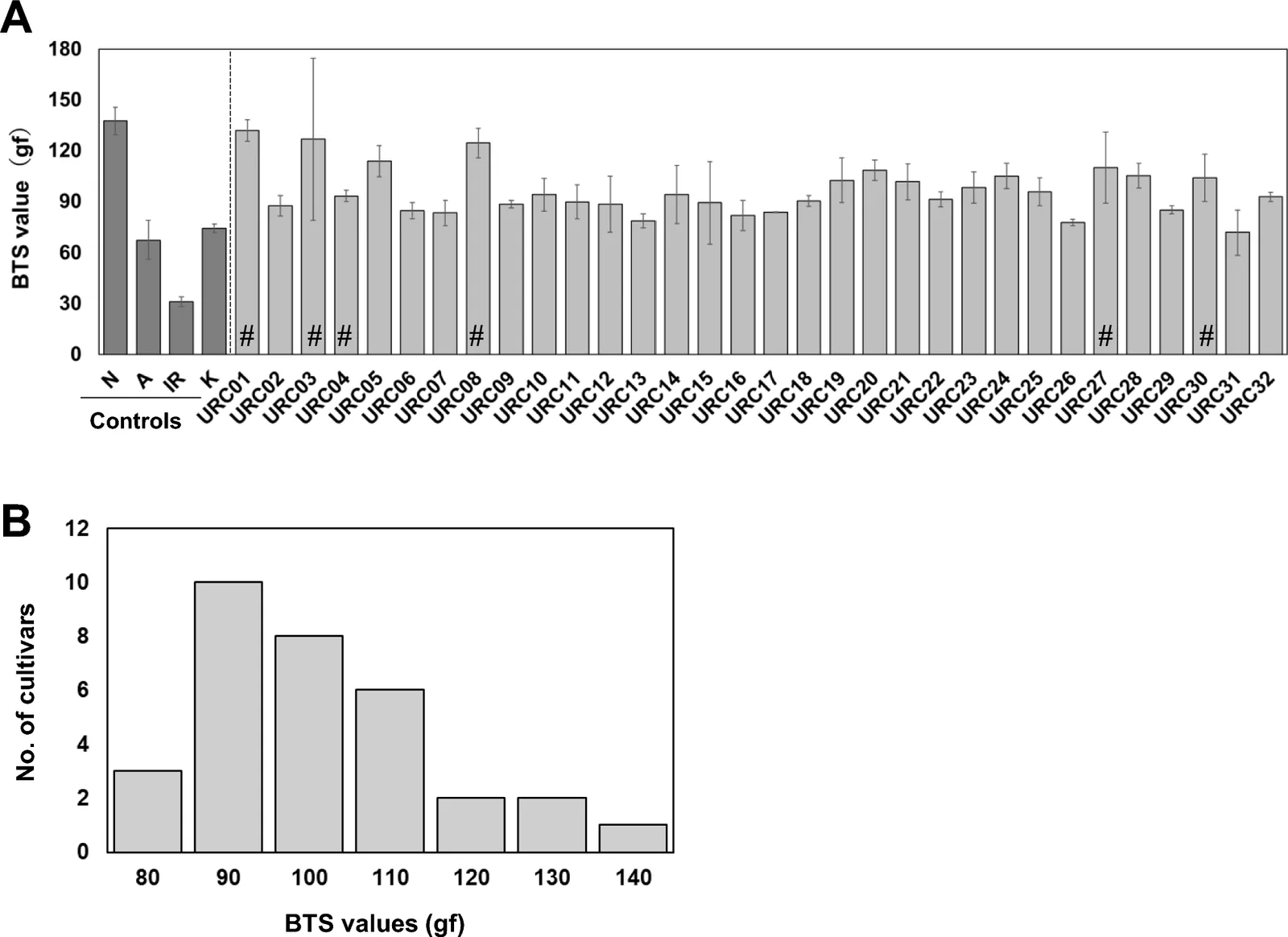
Evaluation of the degree of seed shattering in the Japanese local upland rice core collection. A The degree of seed shattering was estimated by measuring breaking tensile strength (BTS) values for 32 cultivars and control plants; N: Nipponbare, A: Asahi, IR: IR36, K: Kasalath. Data are presented as mean ± SD (n = 4). Hash symbols (#) indicate URC cultivars carrying the N-N-N genotype at the three seed-shattering loci (*qSH1*, *sh4*, and *qSH3*), with reference to Table 2. A significant difference (*p* < 0.01) in BTS values was observed between the URC cultivars with (N–N-N) and without (F-N-N) the hash symbol. B Frequency distribution of BTS values for 32 URC cultivars

All 32 URC cultivars, together with the control cultivars of Nipponbare, Asahi, IR36, and Kasalath, were grown in paddy field, and their panicles were harvested at least one month after heading to minimise the potential confounding effect of seed maturation time on BTS measurements (Fig. 1). The BTS values of control cultivars Nipponbare, Asahi, IR36, and Kasalath were 137.6 ± 8.1 gf, 67.5 ± 11.3 gf, 31.1 ± 2.8 gf, and 74.0 ± 2.4 gf, respectively (Fig. 2A). The average BTS values for URC cultivars ranged from 71.7 ± 13.2 to 131.9 ± 6.3 gf. The highest average BTS value was obtained for URC01, which was similar to that of Nipponbare. The lowest BTS value was obtained for URC31. The frequency distribution of the average BTS values across URC cultivars is shown (Fig. 2B). The wide variation in the average BTS values among URC cultivars suggests the potential involvement of genetic factors in the regulation of the degree of seed shattering.

### Evaluation of awn length in URC cultivars

**Fig. 3:**
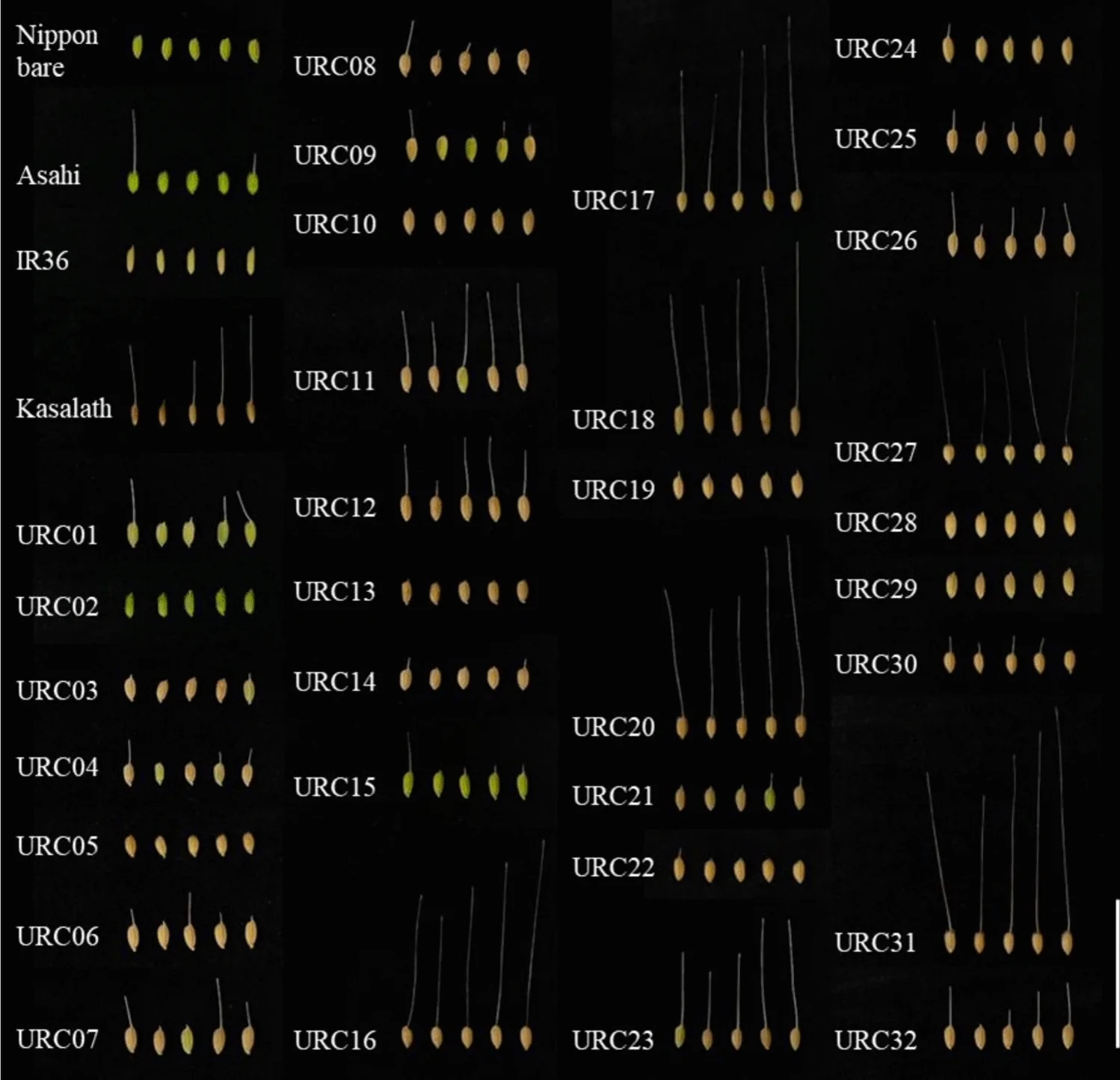
Appearance of awns on the upper first to fifth spikelets. From left to right: first, second, third, fourth, and fifth spikelets. Scale bar = 5 cm

**Fig. 4:**
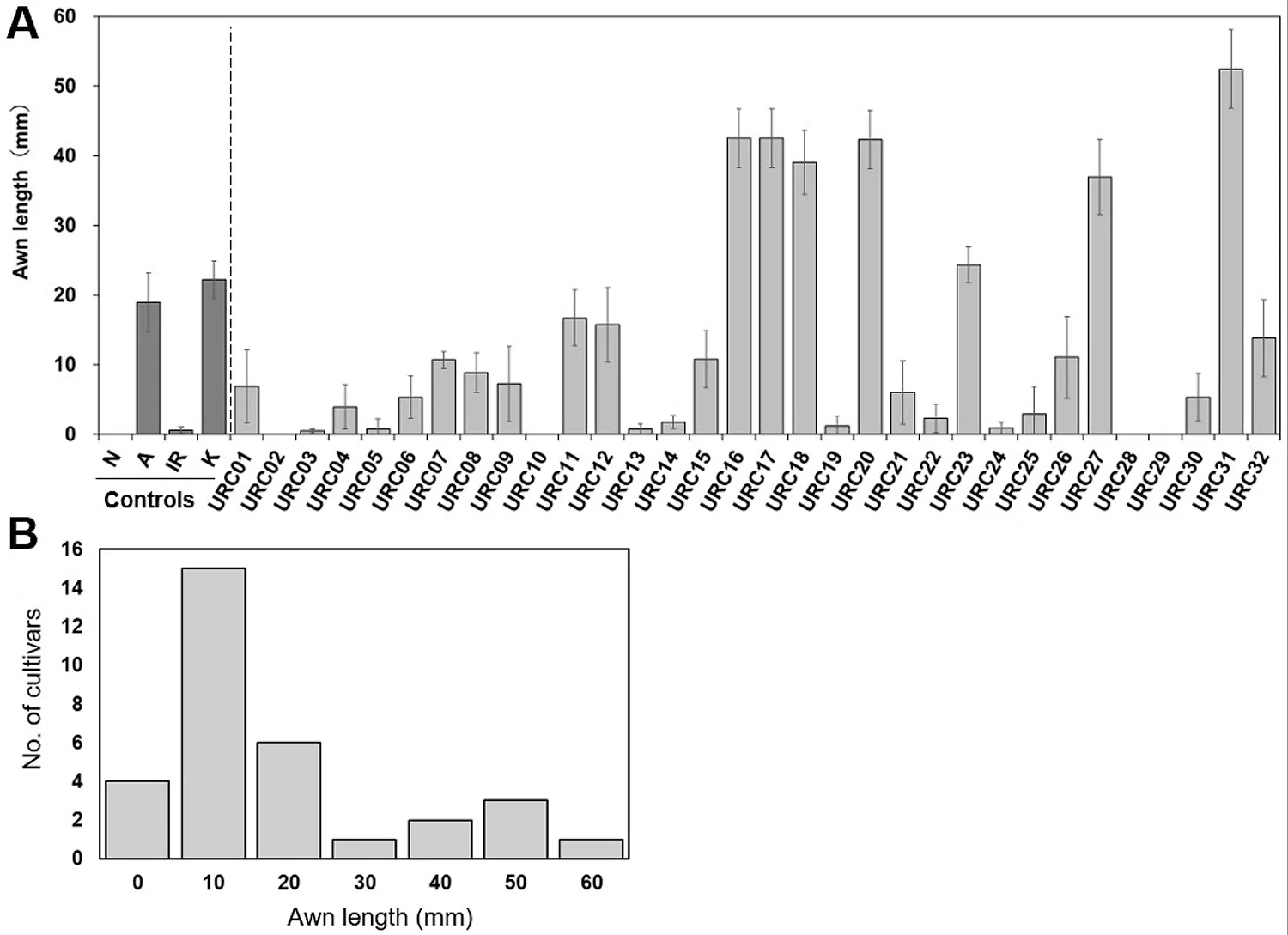
Evaluation of awn length in the Japanese local upland rice core collection. A The average awn length of the first spikelet. Data are presented as mean ± SD (n = 4). B Frequency distribution of awn length for 32 URC cultivars

In the URC, several cultivars were found to possess awns (Figs. 1, 3). To evaluate the degree of awn development in the URC, awn length was measured based on grain position at the heading stage. The top grains tended to be awned, as observed in the cultivars Asahi and Kasalath (Fig. 3). In the first spikelet, variation in mean awn length was observed and URC31 showed the longest awn length (52.5 ± 5.6 mm), whereas URC02, 10, 28, and 29 showed no awn in the top grain (Fig. 4A). The frequency distribution of the average awn length across URC cultivars is shown (Fig. 4B). We also measured the awn length from the second to fifth grains. This variation was similar to that observed in the top grains (Fig. S2). Additionally, we conducted correlation analyses for awn lengths among different spikelet positions on the panicle. Clear positive correlations were observed for awn length among all positions (Table S3).

### Genotyping analyses of the loci controlling seed shattering and awn development in URC cultivars

To better understand the genetic basis of the differences in the degrees of seed shattering and awn length among URC cultivars (Figs. 2, 4), three seed-shattering loci, *qSH1*, *sh4*, and *qSH3*, as well as three awning loci, *An-1*, *LABA1*/*An-2*, and *RAE2/GAD1*/*An-3* were analysed (Table 1). To survey genotypes at three seed-shattering loci, dCAPS markers were used to determine the genotypes of the causal single nucleotide polymorphisms (SNPs) (Table S2, Fig. S1). In the control plant, wild rice *O. rufipogon* W630 had functional alleles at the three seed-shattering loci. The *qSH1* mutation (G; functional, T; non-functional) was observed in only Nipponbare, whereas the *sh4* mutation (G; functional, T; non-functional) was observed in all four control cultivars. Mutation at *qSH3* (C; functional, T; non-functional) was observed for Nipponbare, Asahi, and IR36 but not in Kasalath (Table 2, Fig. S3). In all URC cultivars, the mutations at *sh4* and *qSH3* were conserved. A non-functional allele at *qSH1* was found in six cultivars (URC01, 03, 04, 08, 27, and 30), whereas the other URC cultivars carried the functional allele. These results suggest that most URC cultivars carry a functional allele at *qSH1* that is associated with easy-shattering behaviour. Therefore, the reduced degree of seed shattering in URC can be explained primarily by *qSH1*, and possibly by additional loci, but unlikely by *sh4* and *qSH3*.

**Table 1:** Genes associated with seed shattering and awn development analysed in this study

Trait	Gene	Gene code	Causal mutation	References
Seed shattering	*qSH1*	*Os01g0848400*	SNP at regulatory region	Konishi et al. (2006)
	*sh4 / SHA*	*Os04g0670900*	SNP at gene body	Li et al. (2006), Lin et al. (2007)
	*qSH3*	*Os03g0650000*	SNP at gene body	Ishikawa et al. (2022)
Awn formation	*An-1*	*Os04g0350700*	Insetion or Deletion at gene body	Luo et al. (2013)
	*LABA1 / An-2*	*Os04g0518800*	Deletion at gene body	Hua et al. (2015), Gu et al. (2015)
	*RAE2 / GAD1 / An-3*	*Os08g0485500*	Deletion at gene body	Bessho-Uehara et al. (2016), Jin et al. (2016), Liu et al. (2017)

**Table 2:** Summary of genotypes at three seed-shattering and three awning loci in addition to the BTS values and awn length

Accession	Name of variety or accession	Seed shattering				Seed awning			
		*qSH1*^1)^	*sh4*^2)^	*qSH3*^3)^	BTS (gf)	*An-1*^4)^	*LABA1*^5)^	*RAE2*^6)^	Awn length (mm)
Control	W630	F	F	F	N.A.^7)^	F	F	F	N.A.^7)^
	Nipponbare	N	N	N	137.6 ± 8.1	N(Tn +)	F	N(4C)	0
	Asahi	F	N	N	67.5 ± 11.3	N(Tn +)	F	N(4C)	18.9 ± 4.2
	IR36	F	N	N	31.1 ± 2.8	N(G-)	N	F	0.6 ± 0.4
	Kasalath	F	N	F	74.0 ± 2.4	F	F	N(4C)	22.2 ± 2.7
URC 01	BOSO	N	N	N	131.9 ± 6.3	N(Tn +)	F	N(4C)	6.9 ± 5.2
URC 02	SANKANKA	F	N	N	87.5 ± 6.1	N(G-)	N	N(7C)	0
URC 03	CHIKANARI JUN 1	N	N	N	126.7 ± 47.8	N(Tn +)	N	N(7C)	0.5 ± 0.3
URC 04	KURNAI	N	N	N	93.2 ± 3.3	N(Tn +)	N	N(7C)	3.9 ± 3.2
URC 05	MUTSUKI	F	N	N	113.7 ± 9.2	N(Tn +)	N	N(7C)	0.7 ± 1.5
URC 06	YAKAN	F	N	N	84.5 ± 4.7	N(Tn +)	N	N(7C)	5.3 ± 3.0
URC 07	YAMANOI	F	N	N	83.1 ± 7.6	N(Tn +)	N	N(7C)	10.7 ± 1.2
URC 08	SUZUMESHIRAZU	N	N	N	124.5 ± 8.8	N(Tn +)	N	N(7C)	8.9 ± 2.9
URC 09	KAMEJI	F	N	N	88.4 ± 2.3	N(Tn +)	N	N(7C)	7.3 ± 5.4
URC 10	KURUMI WASE 18	F	N	N	93.9 ± 9.7	N(Tn +)	N	N(7C)	0
URC 11	KYUUSHUU	F	N	N	89.7 ± 10.0	N(Tn +)	N	N(7C)	16.7 ± 4.0
URC 12	OOSHIMA 2	F	N	N	88.3 ± 16.5	N(Tn +)	N	N(7C)	15.7 ± 5.3
URC 13	KYUUZOU	F	N	N	78.5 ± 4.1	N(Tn +)	N	N(7C)	0.7 ± 0.8
URC 14	EDATARE BOUZU	F	N	N	94.0 ± 17.2	N(Tn +)	N	N(7C)	1.7 ± 1.0
URC 15	AKA YAKAN	F	N	N	89.4 ± 24.2	N(Tn +)	N	N(7C)	10.8 ± 4.1
URC 16	FUNDE CHAN GOMI	F	N	N	81.5 ± 8.9	F	N	N(7C)	42.5 ± 4.2
URC 17	BANSEI TAROU	F	N	N	83.6 ± 0.3	F	N	N(7C)	42.5 ± 4.2
URC 18	CHOUKEI SHAKUSEN MODOSHI	F	N	N	90.4 ± 3.2	F	N	N(7C)	39.1 ± 4.6
URC 19	ONJO HAKAMURI	F	N	N	102.5 ± 13.2	F	N	N(7C)	1.2 ± 1.4
URC 20	KANEKO MOCHI	F	N	N	108.4 ± 6.0	F	F	F	42.3 ± 4.2
URC 21	OOSHIMA 4	F	N	N	101.6 ± 10.6	N(Tn +)	N	N(7C)	6.0 ± 4.6
URC 22	ESOJIMA	F	N	N	91.2 ± 4.5	N(Tn +)	N	N(7C)	2.3 ± 2.0
URC 23	TAKASAGO WASE	F	N	N	98.2 ± 9.2	N(Tn +)	N	N(7C)	24.3 ± 2.6
URC 24	KINPOUZAKI 43	F	N	N	105.0 ± 7.5	N(Tn +)	N	N(7C)	0.9 ± 0.9
URC 25	TOUKYOU TOUZOU MOCHI	F	N	N	95.5 ± 8.3	N(Tn +)	N	N(7C)	2.9 ± 3.8
URC 26	MINO MOCHI(MOCHI)	F	N	N	77.6 ± 1.9	N(Tn +)	N	N(7C)	11.1 ± 5.9
URC 27	HINOGE MOCHI	N	N	N	110.0 ± 21.0	F	N	F	36.9 ± 5.4
URC 28	YOSHINO MOCHI 114	F	N	N	105.3 ± 7.3	N(Tn +)	N	N(7C)	0
URC 29	KAGOSHIMA GAISEN MOCHI	F	N	N	85.0 ± 2.5	N(Tn +)	N	N(7C)	0
URC 30	KANAGAWA	N	N	N	103.9 ± 13.9	N(Tn +)	N	N(7C)	5.3 ± 3.4
URC 31	BARA BARA	F	N	N	71.7 ± 13.2	F	F	F	52.5 ± 5.6
URC 32	SEYA 2	F	N	N	92.8 ± 2.7	N(Tn +)	N	N(7C)	13.8 ± 5.5

^1)^Genotype at *qSH1*, F; functional, N; non-functional (Konishi et al. 2006)

^2)^Genotype at *sh4*, F; functional, N; non-functional (Li et al. 2006)

^3)^Genotype at *qSH3*, F; functional, N; non-functional (Ishikawa et al. 2022)

^4)^Genotype at *An-1*, F; functional, N(G-) and N(Tn +); non-functional (Luo et al. 2013)

^5)^Genotype at *LABA1*, F; functional, N; non-functional (Hua et al. 2015)

^6)^Genotype at *REA2*, F; functional, N(4C) and N(7C); non-functional (Bessho-Uehara et al. 2016)

^7)^N.A. not available

Next, we surveyed the genotypes at three awning loci: *An-1*, *LABA1*/*An-2*, and *RAE2*/*GAD1*/*An-3* using InDel markers (Table S2, Fig. S1). In the control, wild rice *O. rufipogon* W630 had functional alleles at the three awning loci (Fig. S4). Variations were found at *An-1*, with two major alleles for reducing awn length (Table 2, Fig. S1). Control cultivars have two alleles: those mainly found in *japonica* are designated as N(Tn +), which results in a non-functional allele due to the downregulation of *An-1* gene expression by transposon insertion, and those mainly found in *indica* are designated as N(G-), which results in a non-functional allele due to a 1-bp deletion (Luo et al. 2013). The genotype with elongated awns was designated as F (functional type). Among URC cultivars, URC16, 17, 18, 19, 20, 27, and 31 had the functional allele as observed in Kasalath and W630; URC02 had the N(G-) genotype that is same as IR36; and all other cultivars had the Tn+ genotype as observed in Nipponbare and Asahi (Table 2, Fig. S4). *LABA1*/*An-2* is characterised by functional (F) and non-functional (N; 1-bp deletion) alleles affecting awn elongation (Hua et al. 2015; Gu et al. 2015). Only URC01, 20, and 31 had the functional allele, as observed for Nipponbare, Asahi, Kasalath, and W630, whereas all other URC cultivars had the non-functional allele, as observed for IR36. Regarding *RAE2*/*GAD1*/*An-3*, there are three alleles corresponding to a highly variable cysteine-rich region (Bessho-Uehara et al. 2016). They are shown as 4C/short, 6C/medium, and 7C/long, according to the number of cysteine residues in the cysteine-rich region, which is reflected in the length class of the protein. The functional allele (F) conferred by the 6C type similar to W630 and IR36 in control plants functionally promoted awn elongation, whereas 4C and 7C were non-functional. URC20, 27, and 31 had the functional allele like IR36 and W630, and only URC01 had the non-functional allele of 4C type like Nipponbare, Asahi, and Kasalath (Table 2, Fig. S4). The other URC cultivars carried the non-functional allele of the 7C type, which was not observed in control cultivars. Taken together, the genotyping survey of known seed-shattering and awning loci revealed allelic variations at the three awning loci.

## Discussion

### Genetic basis of reduced seed shattering in URC cultivars

Among the 32 cultivars in URC, the causal mutations at *sh4* and *qSH3* were found to be fixed with previously reported non-functional alleles (Li et al. 2006; Ishikawa et al. 2022; Table 2). The mutation at *sh4* is conserved in all cultivated rice tested to date, whereas the mutation at *qSH3* is conserved in *indica* and *japonica* rice but not in aus cultivars (Ishikawa et al. 2022; Shionari et al. 2025). In our previous study, we showed that mutations at *sh4* and *qSH3* contributed to reduced seed shattering in both *japonica* and *indica* rice (Inoue et al. 2015; Ishikawa et al. 2022; Sugiyama et al. 2023). A recent study on the evolutionary history of the functional mutation at *qSH3* showed that it may have originated in wild rice populations prior to domestication (Alam et al. 2025). Therefore, mutations at *sh4* and *qSH3* are likely prerequisites for further reduction of seed shattering in URC cultivars. Genotyping analysis at *qSH1* showed variation among URC cultivars (Table 2, Fig. S3). The numbers of cultivars carrying functional and non-functional alleles at *qSH1* were 6 and 26, accounting for 18.8% (93.2–131.9 gf; SD=15.1) and 81.3% (71.7–113.7 gf; SD=10.1), respectively. The average BTS values for individuals carrying functional and non-functional alleles were 115.0 and 91.6 gf, respectively. A Student’s t-test confirmed a significant difference in BTS values between these two allelic groups (*p* < 0.01) (Fig. 2). In *O. rufipogon*, a single mutation at *qSH1* results in complete abscission layer formation (Ishii et al. 2013; Htun et al. 2014), whereas mutations at *qSH1* in addition to *sh4* and *qSH3* disrupt abscission layer formation (Tsujimura et al. 2019). Therefore, the accumulation of mutations at several seed-shattering loci underlies the reduced seed shattering in rice. URC cultivars without the *qSH1* mutation showed lower BTS values than those with the mutation; however, there was a large variation in the average BTS values among cultivars carrying the functional allele at *qSH1* (approximately 42.0 gf, i.e. ranging from 71.7 ± 13.2 gf in URC31 and 113.7 ± 9.2 gf in URC05). These findings suggest that other unknown loci may contribute to the variation of seed shattering in URC cultivars. Recent studies have identified novel seed-shattering loci, such as *qCSS3.1* using *japonica* rice Nipponbare (Tsujimura et al. 2019), *qCSS2.1* and *qCSS7.1* using *indica* rice IR36 (Sugiyama et al. 2023), and *qCSS3.2*, *qCSS4.2*, and *qCSS12.1* using aus cultivar Kasalath (Shionari et al. 2025), suggesting that additional loci may also be involved in reduced seed shattering in upland rice. Further genetic studies are required to understand the genetic basis of reduced degree of seed shattering in the upland rice cultivars. If novel loci involved in the reduced seed-shattering behaviour of upland rice are successfully identified, it would be interesting to investigate the alleles at these loci in other rice cultivars.

Our findings reflect that *sh4* and *qSH3* are completely fixed while *qSH1* remains polymorphic (18.8% functional vs. 81.3% non-functional), providing critical insights into the distinct domestication pathway and historical selection pressures experienced by Japanese upland rice. In temperate lowland *japonica* rice, the non-functional *qSH1* allele is prevalent and facilitates mechanical or intensive harvesting. In contrast, the substantial retention of the functional *qSH1* allele in URC cultivars suggests that traditional upland rice farming in Japan may have experienced different selection intensities, potentially owing to primitive harvesting practices or a higher tolerance for moderate seed shattering in rain-fed, non-flooded environments. Furthermore, the significant variation in BTS values among cultivars sharing the identical functional *qSH1* allele strongly suggests the presence of previously unidentified modifier loci or novel alleles unique to upland ecotypes, such as loci identified in other groups of cultivars (Tsujimura et al. 2019; Sugiyama et al. 2023; Shionari et al. 2025). Therefore, evaluating the genetic mosaic of these known and unknown seed-shattering loci within the URC extends beyond the description of phenotypic diversity, and directly serves as a genetic compass to trace the unresolved geographical origins, migration routes, and cultural distribution of upland rice across Japan.

### Genetic basis of reduced awn length in URC cultivars

Regarding awn length in the URC cultivars, three major loci were surveyed (Table 1). The Tn+ allele at *An-1* is mostly unique to both temperate and tropical *japonica*, whereas the G- allele is unique to *indica* (Luo et al. 2013). The non-functional *LABA1*/*An-2* allele is reported to be unique to *indica* and tropical *japonica* (Hua et al. 2015). The *RAE2*-6C type is functional, whereas the -4C and -7C types are non-functional and are frequently detected both in *indica* and tropical *japonica* cultivars (Bessho-Uehara et al. 2016). When the URC cultivars are categorised by their specific allelic combinations at *An-1*, *LABA1*/*An-2*, and *RAE2*/*GAD1*/*An-3* (independent of the seed-shattering trait), the shared genotypes among the accessions become clearly evident, with a major group comprising 23 URC cultivars carrying the identical genotypic combination of Tn+, N, and 7C, respectively (Table 2). This combination shares similarities with those often observed in tropical *japonica* cultivars, reflecting the genetic characteristics of the URC cultivars analysed in this study.

We observed four URC cultivars (i.e. URC02, 10, 28, and 29) with no awn. Previous studies revealed that there was a nonlinear correlation between the average awn lengths and percentages of awned spikelets (Gu et al. 2005; Mispan et al. 2013). Although the genotypes at *LABA1*/*An-2* and *RAE2*/*GAD1*/*An-3* in the four URC cultivars are non-functional, these non-functional alleles are also detected in awned plants. This observation implies that alternative or additional genetic mechanisms potentially influence awn development in these specific cultivars, which warrants further investigation. On the other hand, we observed seven URC cultivars that had long awns of more than 20 mm (i.e. URC16, 17, 18, 20, 23, 27, and 31). Among these cultivars, URC20 and 31 carry functional alleles at the three loci as wild rice. URC16, 17, 18, and 27 carried functional alleles similar to wild rice at *An-1*, even though they carried non-functional alleles at either *LABA1*/*An-2* or *RAE2*/*GAD1*/*An-3* (Table 2), indicating that the *An-1* functional allele has an epistatic role in promoting awn elongation. Interestingly, URC23 has a relatively longer awn (24.3 ± 2.6 mm; Fig. 4), but all three awning loci carry non-functional alleles (Table 2, Fig. S4). As these three loci are major contributors to reduced awn length, URC23 may carry additional mutations that suppress their effects. Therefore, genetic analysis may be useful for detecting these loci and could be explored in future studies using segregating populations derived from crosses between URC23 and other short-awning cultivars carrying the same genotypic combination at the three awning loci. URC19 carried a functional allele at *An-1* and non-functional alleles at both *LABA1*/*An2* and *RAE2*/*GAD1*/*An-3* (Table 2, Fig. S4). It exhibited an extremely short awn (1.2 ± 1.4 mm; Table 2, Fig. 4), which was much shorter than that of the other three URC cultivars carrying the same alleles at the three awning loci (URC16, 17, and 18). This suggests that URC19 carries other mutations at distinct loci. Genetic analyses of awn length using segregating populations between the URC cultivars will also be of interest for understanding the genetic basis of awn development in rice.

Crucially, our finding that most URC cultivars (23 out of 32, 71.9%) share the specific allelic combination characteristic of tropical *japonica* provides a pivotal genetic clue toward elucidating the historical origin and distribution of upland rice in Japan. While modern temperate *japonica* cultivars in Japan are predominantly awnless due to rigorous selection during modern breeding, the strong genetic affinity between Japanese local upland rice and tropical *japonica* subgroups suggests that upland rice may have been introduced to Japan via a distinct migratory route, potentially originating from Southeast Asia or the southern islands, rather than through the primary route associated with the introduction of lowland temperate *japonica*. Furthermore, the coexistence of fixed non-functional alleles at *sh4* and *qSH3* alongside highly variable awn length phenotypes (ranging from awnless to more than 20 mm) reflects a unique post-domestication selection pressure in Japanese upland ecosystems. In the early phase of rice domestication, longer awns played a critical role in mechanically stabilising panicle structures by supporting grains in panicles with a closed panicle trait regulated by *SPR3* (Ishii et al. 2013; Ishikawa et al. 2022). Although the selection of non-shattering traits eventually rendered long awns evolutionarily redundant for seed dispersal (Ishii and Ishikawa 2018), the evolutionary persistence of long awns in several Japanese upland landraces highlights their practical agronomic advantages in traditional rain-fed farming. Long awns potentially provide protection against bird and animal foraging (Suganami et al. 2024) in rice cultivars growing in upland fields, which are ecologically more vulnerable to wildlife than flooded lowland cultivars, or played an adaptive role in microclimate regulation, such as moisture interception in drought-prone environments. Thus, the architectural mosaic of genotypes in the URC demonstrates that the reduction of awns in Japanese upland rice was not a uniform evolutionary event, but rather a dynamic process reflecting a balance between general domestication trends and localised agro-ecological demands.

Although this study provides valuable insights into the phenotypic variations for seed shattering and awn length, the limited sample size precluded us from performing a robust genome-wide association analysis. Consequently, further genetic studies with a larger population and high-density genotyping are warranted to elucidate the precise genotype–phenotype associations, as modelled by previous studies (Gu et al. 2005; Mispan et al. 2013). Such future genetic insights will undoubtedly enhance the annotation and functional interpretation of our phenotypic dataset, and further delineate the precise genetic basis underlying the maintenance or modification of these traits during the expansion of upland rice cultivation across Japan.

### Utility of the URC as a resource for domestication studies and future rice breeding

Among the 32 URC cultivars, large variations in the degrees of seed shattering and especially in awn length were observed (Figs. 2, 4), and the genotypes at the loci responsible for the two traits were surveyed using DNA markers (Table 2). Beyond these domestication-related traits, glutinosity, which indicates eating quality and cultural preferences, serves as another critical determinant of rice distribution. Rice glutinosity is determined by a recessive mutation at the *Waxy* locus that encodes granule-bound starch synthase I (GBSSI), the enzyme responsible for amylose synthesis (Wang et al. 1995). Hybridisation of glutinous cultivars with non-glutinous ones results in a non-glutinous phenotype in the F_1_ hybrids. Consequently, traditional farmers must have managed glutinous cultivars with strict spatial or temporal isolation to maintain this trait, effectively creating a distinct genetic and cultural bottleneck during their historical cultivation. Previously, Okano et al. (2006) reported glutinosity in 11 (34.4%) URC cultivars. In this study, we confirmed the high frequency of glutinosity based on iodine staining and by genotyping the causal 23-bp insertion at the *Waxy* locus (Table S4, Fig. S5), demonstrating a clear genotype–phenotype association. Overall, the genetic mosaic in the URC, as characterised by the incomplete fixation of non-shattering alleles, the predominance of tropical *japonica*-type awning genotypes, and an exceptionally high proportion of glutinous cultivars, strongly suggests that Japanese local upland rice was characterised by a highly specialised distribution and cultural trajectory that likely reflects the influence of traditional rain-fed farming practices and unique culinary preferences, rather than the major historical expansion of modern temperate lowland rice in Japan.

In Japan, the commercial cultivation of upland rice declined rapidly due to economic constraints; however, the URC remains an indispensable bio-resource for evolutionary studies and future rice breeding. Because the URC was established based on morphological and physiological traits without prior genomic data (Okano et al. 2006), the whole-genome sequencing analysis is the ideal next step to uncover hidden genetic variations. From cultural and evolutionary perspectives, the URC extends beyond a description of phenotypic diversity; it functions as a highly concentrated genetic archive that can help resolve the geographical origins and migration routes of upland rice across Japan. Practically, the immense phenotypic variation within this minimal set of accessions makes it a highly efficient toolkit for screening disease resistance and environmental tolerance. Cultivars associated with such agronomic advantages can potentially be introgressed into elite lowland rice via backcrossing. When utilising URC lines for new cultivar development, the diagnostic DNA markers validated in this study for seed-shattering and awning loci will help avoid undesirable genotypic combinations. In conclusion, this comprehensive evaluation of the URC successfully bridges the gap between historical domestication studies and the future agricultural sustainability, highlighting the profound value of preserving local upland rice germplasm.

## Supplementary Information

Below is the link to the electronic supplementary material.

Supplementary file1 (PDF 1502 KB)

## Data Availability

Additional data are provided in Supplementary Tables and Figures.
