# Supplementary material for: Genetic variation in seed shattering and awn length in a core collection of Japanese local upland rice, Oryza sativa L
Source: Genetica. 2026 Nov 6;154(1):54. doi: 10.1007/s10709-026-00300-8 (PMC13743659; doi:10.1007/s10709-026-00300-8)
Supplement: Supplementary file 1 [file 10709_2026_300_MOESM1_ESM.pdf]

Mikumo & Yabu et al. Table S1

Table S1. List of 32 cultivars in Japanese local upland rice core collection (Okano et al. 2006)

ID	Cultivar	Origin
URC 01	BOSO	FOREIGN (Unspecified)
URC 02	SANKANKA	JAPAN (Unspecified)
URC 03	CHIKANARI JUN 1	JAPAN (YAMAGATA)
URC 04	KURNAI	FOREIGN (Unspecified)
URC 05	MUTSUKI	JAPAN (Unspecified)
URC 06	YAKAN	JAPAN (KAGOSHIMA)
URC 07	YAMANOI	JAPAN (GUNMA)
URC 08	SUZUMESHIRAZU	JAPAN (AOMORI)
URC 09	KAMEJI	JAPAN (SHIMANE)
URC 10	KURUMI WASE 18	JAPAN (TOHOKU, Unspecified)
URC 11	KYUUSHUU	JAPAN (KYUSHU, OKINAWA, Unspecified)
URC 12	OOSHIMA 2	JAPAN (KANTO TOSAN, Unspecified)
URC 13	KYUUZOU	JAPAN (Unspecified)
URC 14	EDATARE BOUZU	JAPAN (Unspecified)
URC 15	AKA YAKAN	JAPAN (KAGOSHIMA)
URC 16	FUNDE CHAN GOMI	FOREIGN (Unspecified)
URC 17	BANSEI TAROU	JAPAN (Unspecified)
URC 18	CHOUKEI SHAKUSEN MODOSHI	JAPAN (Unspecified)
URC 19	ONJO HAKAMURI	JAPAN (KYUSHU, OKINAWA, Unspecified)
URC 20	KANEKO MOCHI	JAPAN (TOKYO)
URC 21	OOSHIMA 4	JAPAN (KANTO TOSAN, Unspecified)
URC 22	ESOJIMA	JAPAN (TOCHIGI)
URC 23	TAKASAGO WASE	JAPAN (Unspecified)
URC 24	KINPOUZAKI 43	JAPAN (Unspecified)
URC 25	TOUKYOU TOUZOU MOCHI	JAPAN (TOKYO)
URC 26	MINO MOCHI(MOCHI)	JAPAN (GIFU)
URC 27	HINOGE MOCHI	JAPAN (SHIMANE)
URC 28	YOSHINO MOCHI 114	JAPAN (GIFU)
URC 29	KAGOSHIMA GAISEN MOCHI	JAPAN (KAGOSHIMA)
URC 30	KANAGAWA	JAPAN (KANAGAWA)
URC 31	BARA BARA	JAPAN (Unspecified)
URC 32	SEYA 2	JAPAN (Unspecified)

Mikumo & Yabu et al. Table S2

Table S2. Primers used for genotyping at loci controlling seed shattering and awning in the Japanese local upland rice core collection

Trait	Target	Forward (5'-3')	Reverse (5'-3')	Restriction Emzyme	Reaction condition (° C)
Seed shattering	*qSH1*-causal SNP (dCAPS)	TGATGGTATTGATGTATACTGGACAaT[1)]	TCGAAATGTGGAGACAGCTC	*Eco*T22 (*Ava*III)	37
	sh4-causal SNP (dCAPS)	AACGCCGGCGCGGCGGTCGTCGTCCAGtCG[1)]	ACGGGCACCTGACTGCTAC	*Taq*I	65
	qSH3-causal SNP (dCAPS)	TACGTGCACTGCAACTTCTGCAACAgAAT[1)]	GCAAATGAACTGCCCAAATC	*Eco*RI	37
Awn formation	*An-1*-1bp deletion (nested)[2)]	CGATCTCCACTGATCACCAA	CCCTCGCATTGTGATTAATTT	-	-
	An-1-1bp deletion	CCGACGCGGCGGAGGAGAAG	TCACCCTCTCGGCGAGGCTATGG	-	-
	An-1(Tn)-In	GGTTATTTGCTCCAGGGTCA	GGGAGAGTGGAGGAGGAGAG	-	-
	An-1(Tn)-Out	CCACCCACCAATACGGTTAC	GGGAGAGTGGAGGAGGAGAG	-	-
	LABA1-causal deletion	GAGGGAGAGGCAGTAGTGGA	AGCTCCTTGCCAAGCTCAAC	-	-
	RAE2- causal deletion	CAGGTGCCCACCTTGTCC	AGGGTCAGGGGTCGAACAG	-	-
Glutinosity	*Waxy*- causal insertion	TGCAGAGATCTTCCACAGCA	GCTGGTCGTCACGCTGAG	-	-

[1)] Nucleotide with a small letter indicates the primer mismatch creating restriction endonuclease-sensitive polymorphism.

[2)] These primers were used to detect a 1-bp deletion via nested PCR for *An-1*-1bp deletion.

Mikumo & Yabu et al. Table S3

Table S3. Summary of correlation coefficients (*r*) between the degree of seed shattering and awn length at five spikelet positions in URC

	Trait[1]					
	SH[2]	Awn length based on spiklet position				
		1st	2nd	3rd	4th	5th
SH		0.167	0.155	0.144	0.173	0.177
1st	-0.250		**<0.001**	**<0.001**	**<0.001**	**<0.001**
2nd	-0.257	0.984		**<0.001**	**<0.001**	**<0.001**
3rd	-0.264	0.990	0.995		**<0.001**	**<0.001**
4th	-0.247	0.989	0.992	0.995		**<0.001**
5th	-0.245	0.990	0.992	0.992	0.995	

[1] Values below and above the diagonal represent *r* values and their probability (*P*) levels, respectively.

Values significant at $p < 0.001$ are indicated in bold.

[2] SH, the degree of seed shattering evaluated by BTS values.

Mikumo & Yabu et al. Table S4

Table S4. Genotypes at the *Waxy* locus in the Japanese local upland rice core collection

ID	Cultivar name	Glutinesity	Genotype[1)]
URC 01	BOSO	non-glutinous	F
URC 02	SANKANKA	non-glutinous	F
URC 03	CHIKANARI JUN 1	non-glutinous	F
URC 04	KURNAI	non-glutinous	F
URC 05	MUTSUKI	non-glutinous	F
URC 06	YAKAN	non-glutinous	F
URC 07	YAMANOI	non-glutinous	F
URC 08	SUZUMESHIRAZU	non-glutinous	F
URC 09	KAMEJI	non-glutinous	F
URC 10	KURUMI WASE 18	non-glutinous	F
URC 11	KYUUSHUU	non-glutinous	F
URC 12	OOSHIMA 2	non-glutinous	F
URC 13	KYUUZOU	non-glutinous	F
URC 14	EDATARE BOUZU	non-glutinous	F
URC 15	AKA YAKAN	non-glutinous	F
URC 16	FUNDE CHAN GOMI	non-glutinous	F
URC 17	BANSEI TAROU	non-glutinous	F
URC 18	CHOUKEI SHAKUSEN MODOSHI	non-glutinous	F
URC 19	ONJO HAKAMURI	non-glutinous	F
URC 20	KANEKO MOCHI	non-glutinous	F
URC 21	OOSHIMA 4	glutinous	N
URC 22	ESOJIMA	glutinous	N.A.[2)]
URC 23	TAKASAGO WASE	glutinous	N
URC 24	KINPOUZAKI 43	glutinous	N
URC 25	TOUKYOU TOUZOU MOCHI	glutinous	N
URC 26	MINO MOCHI(MOCHI)	glutinous	N
URC 27	HINOGE MOCHI	glutinous	N
URC 28	YOSHINO MOCHI 114	glutinous	N
URC 29	KAGOSHIMA GAISEN MOCHI	glutinous	N
URC 30	KANAGAWA	glutinous	N
URC 31	BARA BARA	non-glutinous	F
URC 32	SEYA 2	glutinous	N

[1)] Genotype at *Waxy* locus was analysed by primers shown in Table S2.

[2)] PCR fragment was not amplified well.

Mikumo & Yabu et al. Fig. S1

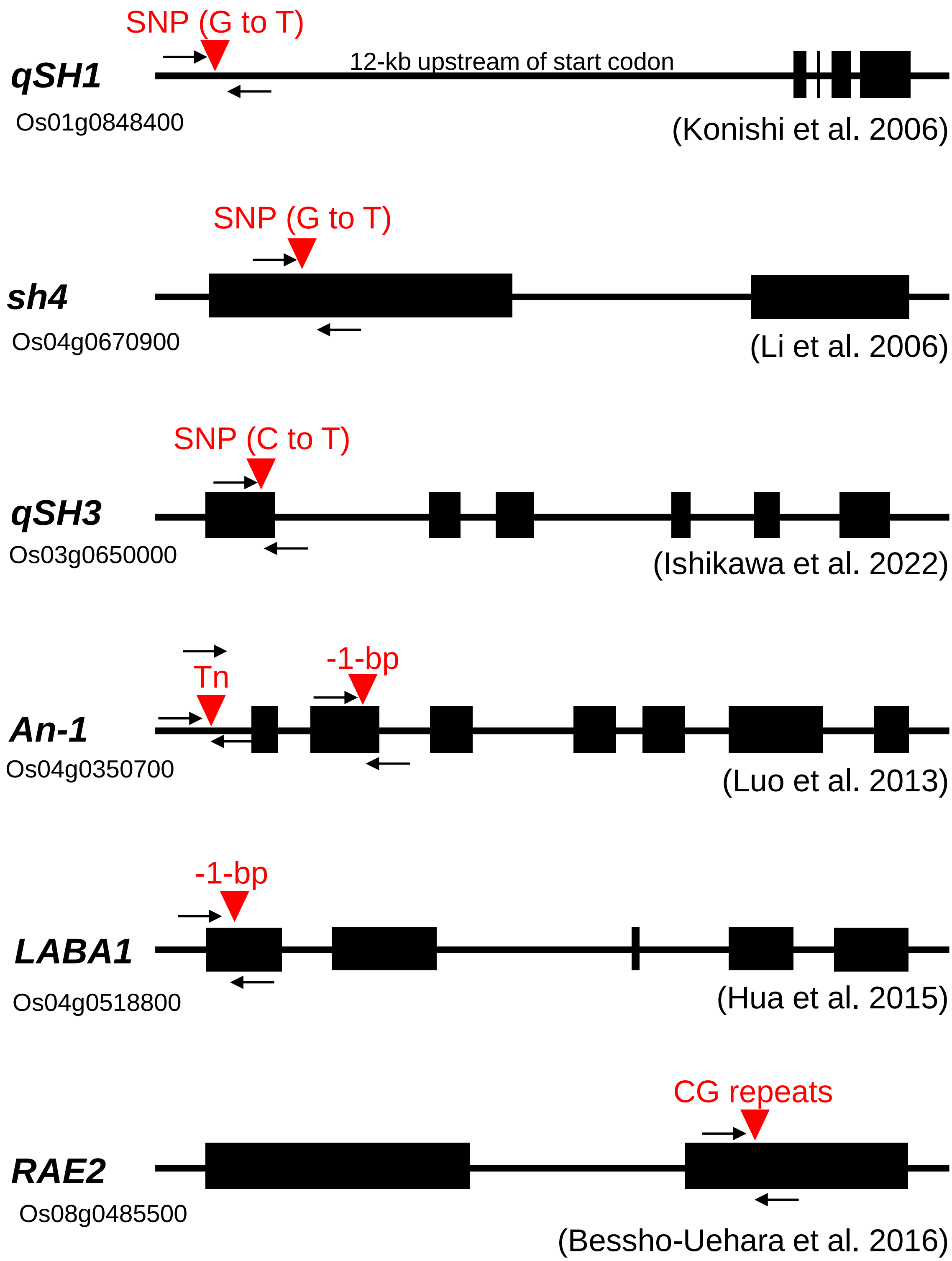

Fig. S1. Schematic representations of three seed-shattering and three awn loci, along with their causal mutations selected during rice domestication. Specific primers were designed to detect the polymorphisms between functional and non-functional alleles. For *An-1*, the loss-of-function allele caused by a Tn insertion was determined using two pairs of primers.

Mikumo & Yabu et al. Fig. S2

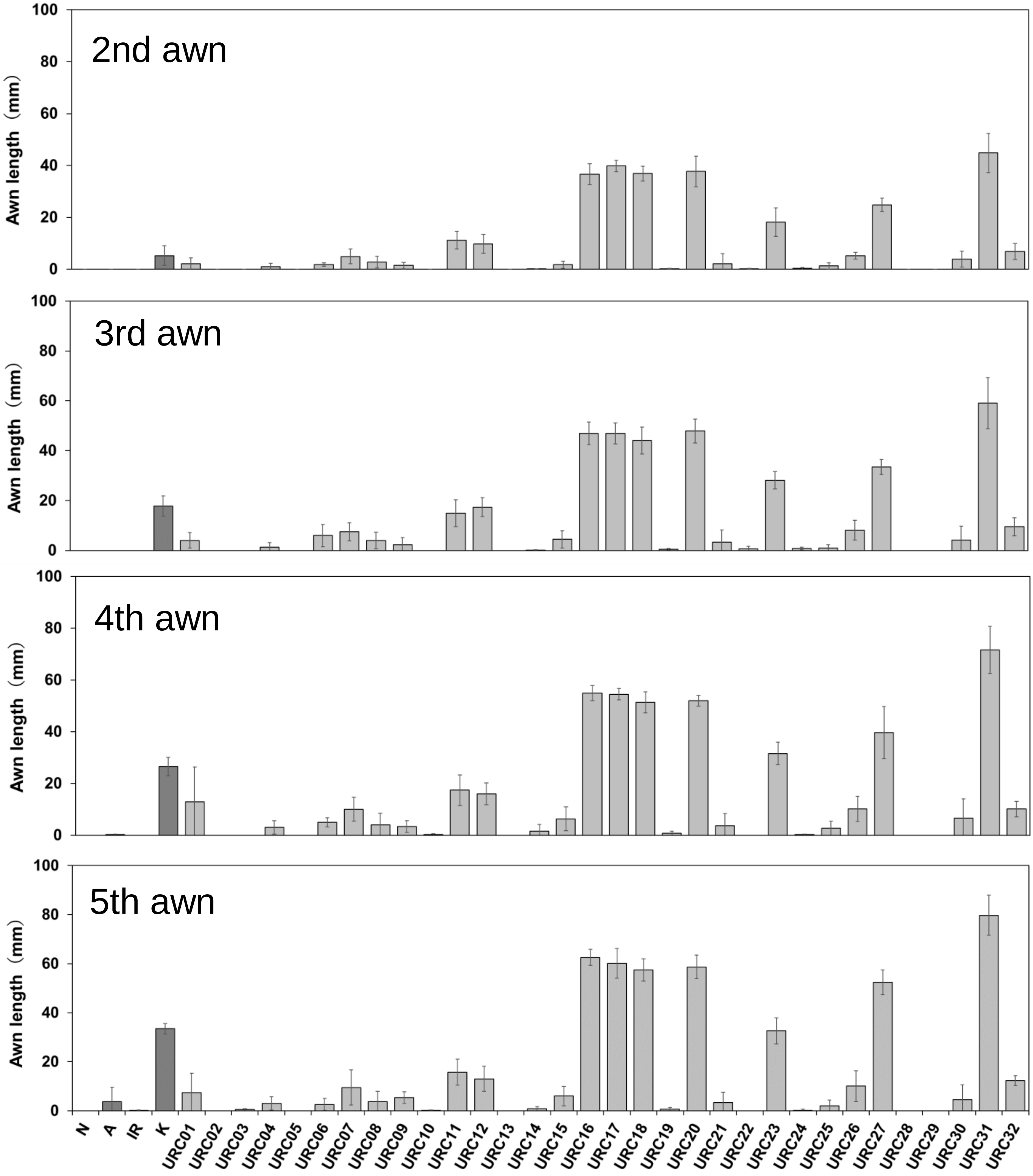

Fig. S2. Evaluation of awn length in the Japanese local upland rice core collection. The awns of the upper 2nd to 5th spikelets are shown.

Mikumo & Yabu et al. Fig. S3.

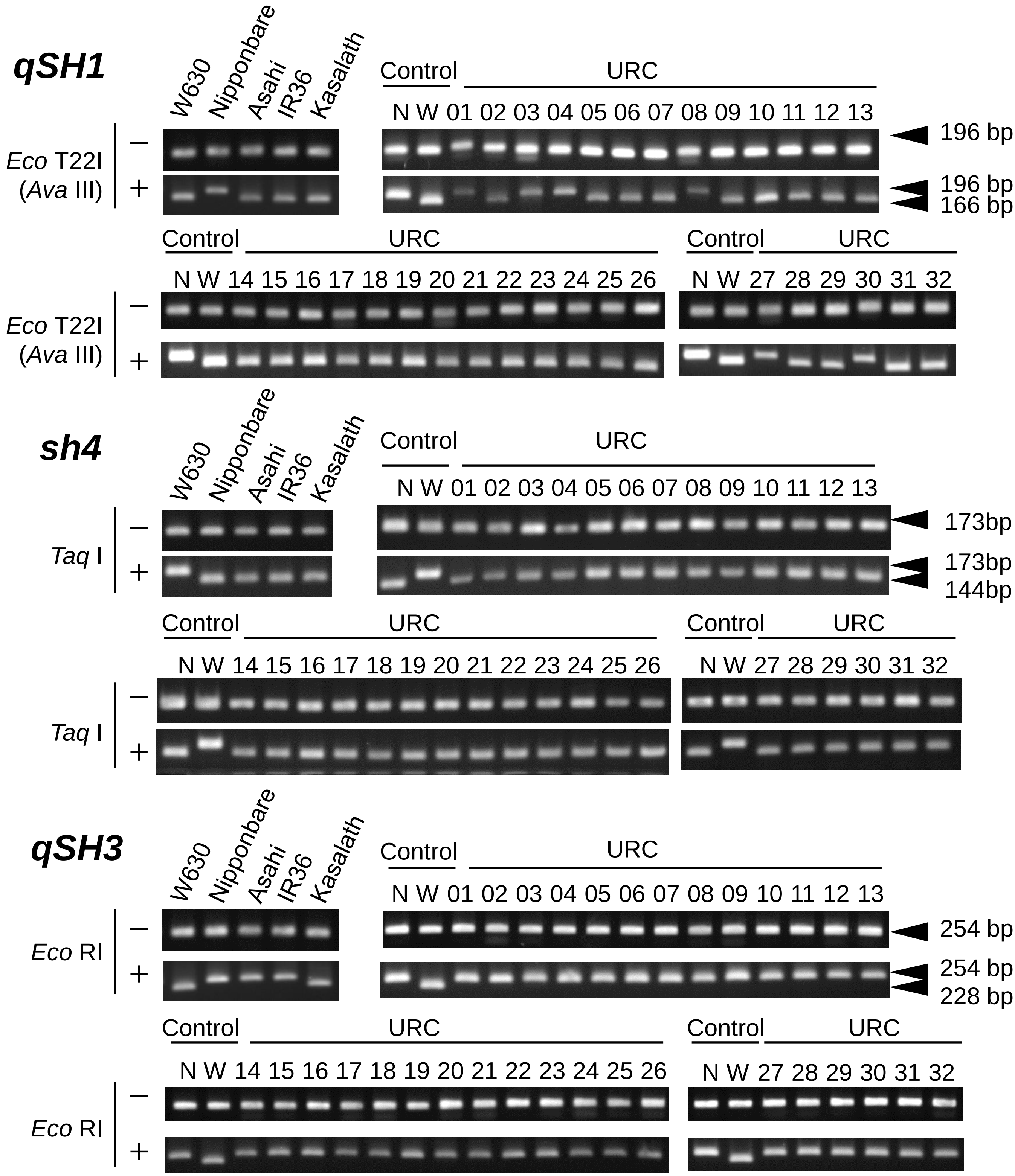

Fig. S3. Genotyping survey for three seed-shattering loci, *qSH1*, *sh4*, and *qSH3* in URC cultivars. Genotyping analyses were conducted at *qSH1*, *qSH3*, and *sh4* for *O. rufipogon* acc. W630 (W), *O. sativa japonica* cv. Nipponbare (N), cv. Asahi, cv, IR36, cv. Kasalath and URC cultivars based on the causal single-nucleotide polymorphisms (SNPs) detected using derived cleaved amplified polymorphic sequence (dCAPS) markers (Table S2). '−' and '+' indicate PCR fragments undigested and digested with specific enzymes, respectively.

Mikumo & Yabu et al. Fig. S4

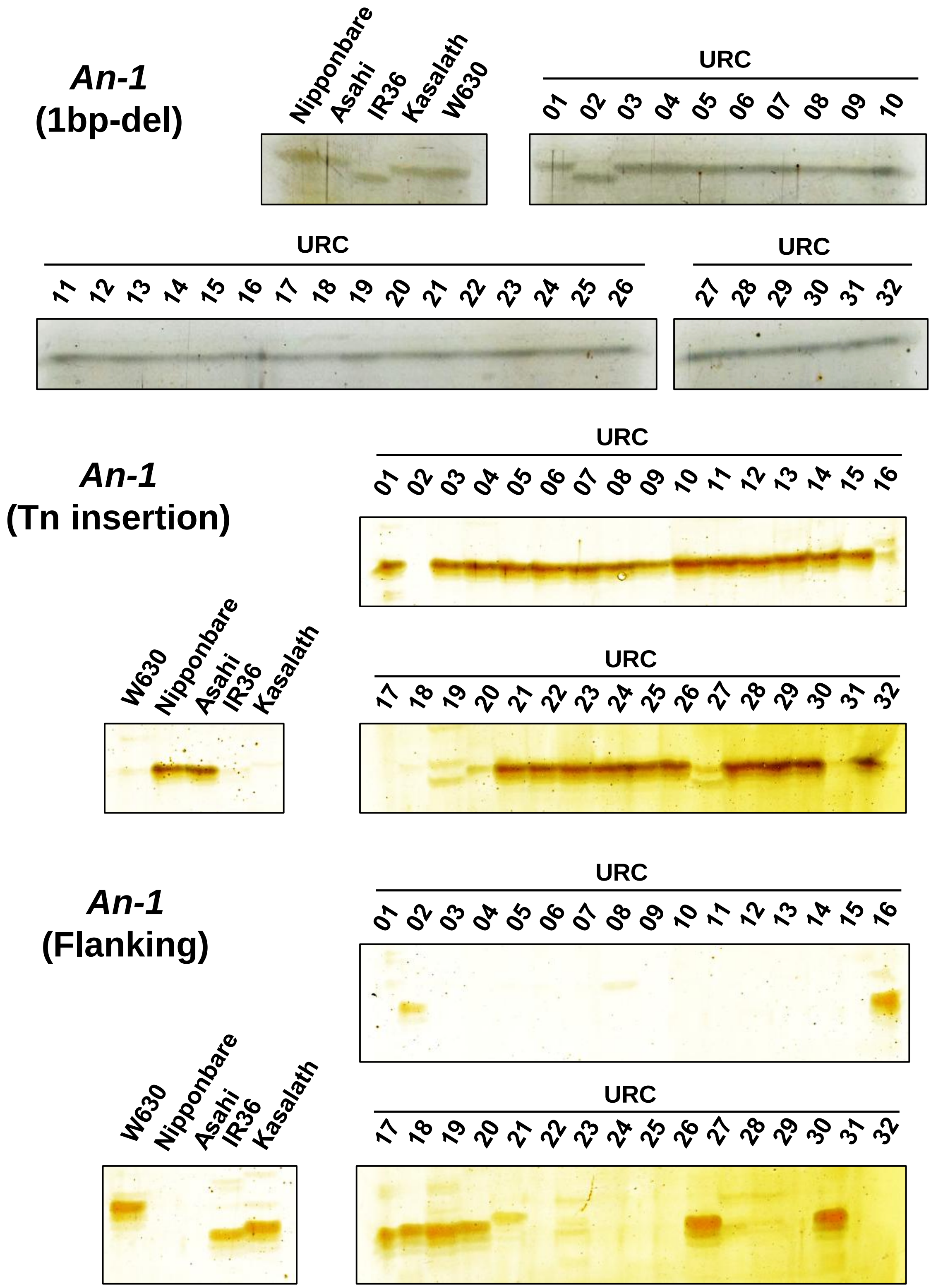

Fig. S4. Genotyping survey for three awning loci, *An-1*, *LABA1*, and *RAE2* in URC cultivars. Genotyping analyses were conducted at *An-1*, *LABA1*, and *RAE2* for *O. sativa japonica* cv. Nipponbare, cv. Asahi, cv. IR36, cv. Kasalath, *O. rufipogon* acc. W630, and URC cultivars based on the Indel markers (Table S2).

Mikumo & Yabu et al. Fig. S4 (continued)

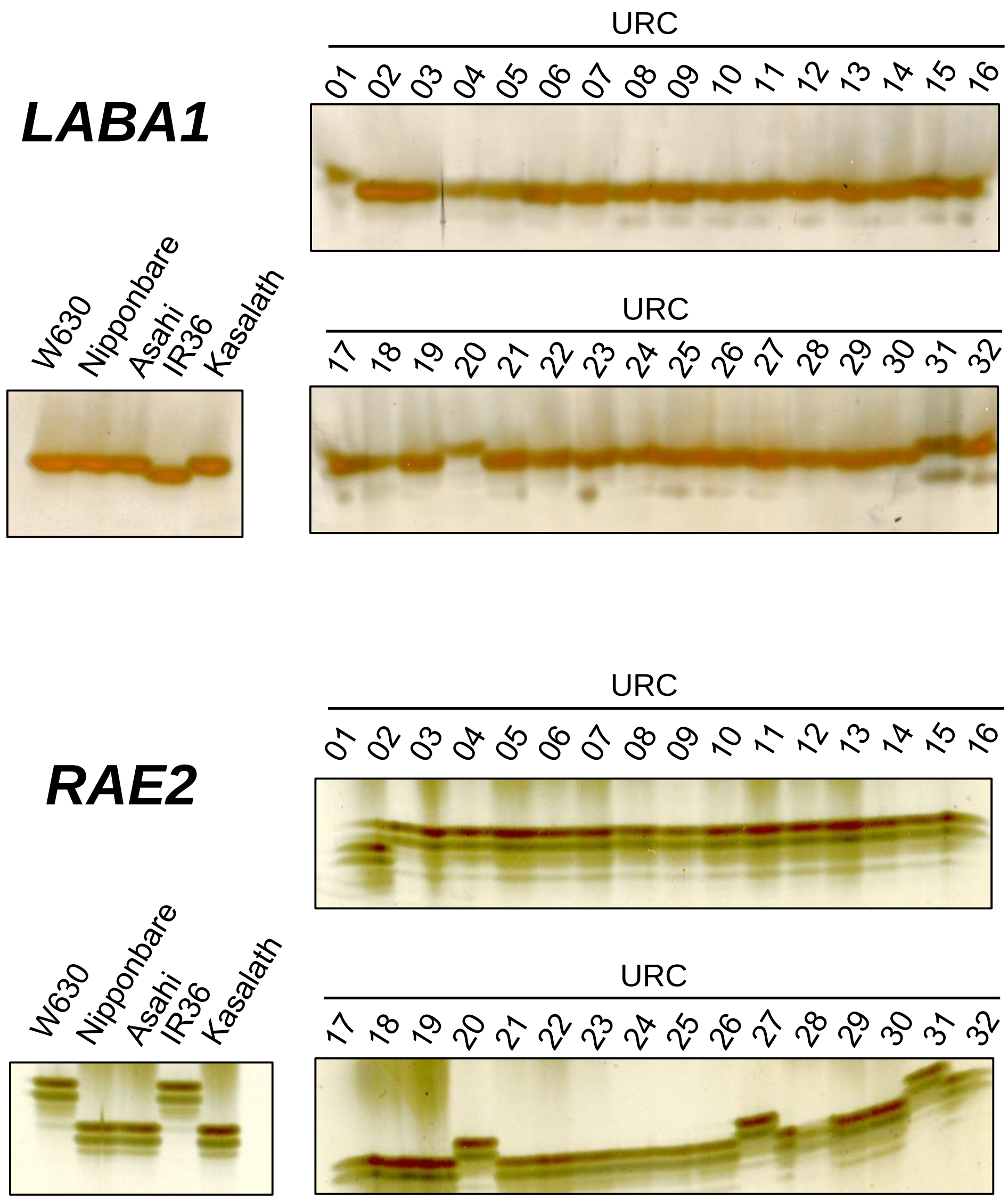

Fig. S4 (Continued). Genotyping survey for three awning loci, *An-1*, *LABA1*, and *RAE2* in URC cultivars.

Genotyping analyses were conducted at *An-1*, *LABA1*, and *RAE2* for *O. sativa japonica* cv. Nipponbare, cv. Asahi, cv. IR36, cv. Kasalath, *O. rufipogon* acc. W630, and URC cultivars based on the InDel markers (Table S2).

Mikumo & Yabu et al. Fig. S5

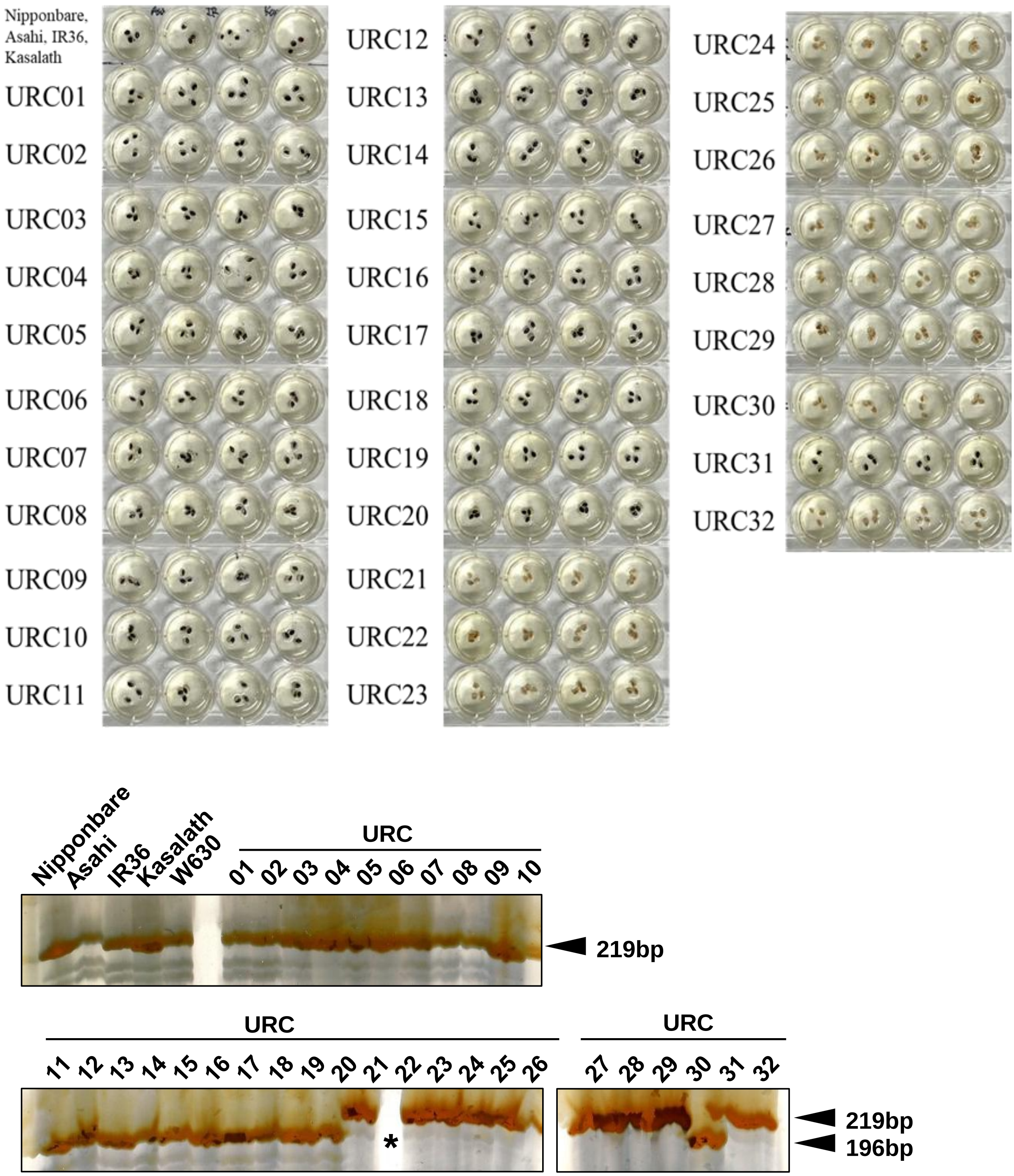

Fig. S5. Evaluation of glutinosity and genotyping survey at the *Waxy* locus in URC cultivars. Results of iodine-starch test for glutinosity (upper panel). Each control was tested using seeds from a single plant. Controls from left to right: Nipponbare, Asahi, IR36, and Kasalath. Genotyping survey for 23-bp InDel polymorphism at *Waxy* (lower panel). *PCR fragment was not amplified in URC22.
